# Preparation Methods and Perceived Risk of Foodborne Illness Among Consumers of Prepackaged Frozen Vegetables – United States, September 2022

**DOI:** 10.1016/j.jfp.2024.100440

**Published:** 2024-12-20

**Authors:** Michelle Canning, Michael Ablan, Tamara N. Crawford, Amanda Conrad, Alexandra Busbee, Misha Robyn, Katherine E. Marshall

**Affiliations:** 1 Division of Foodborne, Waterborne, and Environmental Diseases, National Center for Emerging and Zoonotic Infectious Diseases, Centers for Disease Control and Prevention. 1600 Clifton Rd NE MS-H24-10, Atlanta, GA 30333, USA; 2 Oak Ridge Institute for Science and Education, 1299 Bethel Valley Rd, Oak Ridge, TN 37830, USA

**Keywords:** Foodborne illness, *Salmonella*, *Listeria monocytogenes*, *Escherichia coli*, Frozen vegetables, Consumers

## Abstract

*Listeria monocytogenes* causes listeriosis, a serious infection with a high mortality rate for persons at higher risk for listeriosis. The first *Listeria* outbreak linked to frozen vegetables occurred in 2016 and resulted in three deaths. Many frozen vegetables are intended to be consumed after cooking. However, data on consumer behavior are sparse. We characterized consumers’ perceptions of contamination of prepackaged frozen vegetables, and preparation methods of prepackaged frozen vegetables to help inform prevention strategies. During September 1–24, 2022, Porter Novelli Public Services conducted the FallStyles survey using the Ipsos KnowledgePanel. Data were weighted to be representative of the U.S. population. Point estimates and 95% CIs were calculated, and differences between respondents were determined using Wald chi square tests. Among 3,008 respondents reporting a preparation and consumption method for frozen vegetables, 8.7% (95% CI = 7.4–10.0%) reported ever consuming the product raw. Respondents who reported having children < 18 years old were more likely to report ever consuming frozen vegetables raw compared with respondents who did not (12.5% vs. 7.4%, *p* < 0.01). The most reported raw preparation method was adding them directly to a blender for smoothie or juice (5.6%; 95% CI = 4.6–6.7%). Among respondents who reported eating frozen vegetables, 59.6% (95% CI = 57.6–61.6%) reported following package instructions. A third (34.1% [95% CI = 32.2–35.9%]) of respondents agreed that frozen vegetables can be contaminated with germs (like *Salmonella*, *E. coli*, and *Listeria*), with a greater proportion of people with cancer disagreeing compared to those without cancer (32.5% vs 23.4%, *p* = 0.041). These findings show that some consumers may not be cooking frozen vegetables before eating them. Second, consumers might not be reading instructions on packaging. Both findings highlight the critical importance of preventive controls in the production of frozen vegetables prior to reaching the consumer.

CDC estimates nontyphoidal *Salmonella* causes over one million domestically acquired foodborne illnesses per year in the United States, while Shiga toxin-producing *Escherichia coli* (STEC) causes nearly 176,000 and *Listeria monocytogenes* causes nearly 1,600 (Scallan et al., 2011). *Salmonella* and STEC infections are more likely to occur among children under five, and *Listeria monocytogenes* infections are more likely to occur among adults 65 years and older, people who have a weakened immune system, and people who are pregnant (U.S. Centers for Disease Control and Prevention, 2022a, 2022d). CDC estimates people who are pregnant are 10 times more likely to get a *Listeria* infection than other people (U.S. Centers for Disease Control and Prevention, 2022c).

Vegetables are an important part of a balanced diet. However, contaminated vegetables have also been the source of outbreaks of *Salmonella*, STEC, and *Listeria* infections in the United States. According to the interagency food safety analytics collaboration (IFSAC) estimates, foodborne illnesses have consistently been attributed to vegetables; in 2020, 28.4% of *Salmonella* illnesses, 63.4% of *E. coli* O157 illnesses, and 29.3% of *Listeria monocytogenes* illnesses were attributed to seeded vegetables, sprouts, vegetable row crops, and other produce (which may also include vegetables) (Interagency Food Safety Analytics Collaboration, 2022). In 2016, a listeriosis outbreak was linked to frozen vegetables for the first time in the United States and resulted in the recall of approximately 450 products (U.S. Centers for Disease Control and Prevention, 2016; Madad et al., 2023).

While freezing can help to preserve foods and inhibit bacterial growth, the freezing process does not destroy bacteria like *Listeria monocytogenes*, *Salmonella*, and STEC (Chaves et al., 2011; Muller et al., 2012; Kataoka et al., 2017). Further, upon thawing, bacteria present on food can reproduce again. *Listeria* can reproduce even when food is refrigerated (Kataoka et al., 2017; U.S. Food & Drug Administration, 2017). Therefore, it is important that products that are frozen and refrigerated are prepared and consumed as intended, including any required cooking step (Storey & Anderson, 2018; Willis et al., 2020; American Frozen Food Institute, 2022). However, 11% of respondents in a national survey reported never looking at the cooking instructions for packaged frozen vegetables and 17% responded that they only looked the first time they make a new product (U.S. Food & Drug Administration, 2021). Additionally, research on consumer practices for preparing frozen chicken products suggests a label’s cooking instructions are not always read, that some consumers might not understand the importance of following instructions, and that some consumers might not have the appliances or tools to be able to follow the cooking instructions (Marshall et al., 2022). Thus, relying upon the consumer reading and following labeling with cooking instructions as a primary control measure for reducing bacterial contamination to levels that are unlikely to cause illness could be problematic.

Data collected during outbreak investigations about cooking practices and consumption of frozen vegetables are sparse. While the standard questionnaire used during multistate *Salmonella* or STEC outbreaks (National Hypothesis Generating Questionnaire, NHGQ) does include questions about frozen vegetable consumption, the case report form for all listeriosis cases (*Listeria* Initiative, LI) does not (U. S. Centers for Disease Control and Prevention, 2022b). Neither the NHGQ nor the LI collect information on how consumers prepare frozen vegetables. Because consumers are often expected to fully cook frozen vegetables, more information on the knowledge, attitudes, and practices of consumers toward frozen vegetable preparation and consumption could help inform prevention strategies. The objective of this report is to characterize and compare risk perceptions about frozen vegetables and preparation practices among all consumers, people with weakened immune systems, people who are pregnant, and people aged 65 or older. These data will help to fill current knowledge gaps in how consumers are preparing and consuming prepackaged frozen vegetables and how their perception of the risk of contamination with foodborne pathogens influences their preparation and consumption methods.

## Methods

### Data source.

The FallStyles 2022 survey was conducted by Porter Novelli Public Services from September 1–24, 2022 using the Ipsos KnowledgePanel (an online panel representative of noninstitutionalized U.S. population) (ConsumerStyles & YouthStyles – PNStyles (porternovelli.com)). Respondents aged 18 years and older were recruited by mail utilizing probability-based sampling. The survey was sent to respondents who had previously responded to the SpringStyles survey. If needed, laptops or tablets and internet access were provided for survey completion. This activity was reviewed by CDC and was conducted in a manner consistent with applicable federal law and CDC policy, including 45 C.F.R. part 46; 21 C.F.R. part 56; 42 U.S.C. Sect. 241(d); 5 U.S.C. Sect. 552a; 44 U.S.C. Sect. 3501 et seq.

### Definitions.

To understand and assess the methods respondents use to prepare and cook frozen vegetables, respondents were asked, “How do you prepare and/or consume prepackaged frozen vegetables?”. Response options for this question included several preparation/consumption methods as well as “none of the above” and “I do not eat prepackaged frozen vegetables”. Respondents were able to select as many options as applicable. For bivariate analyses of preparation and consumption methods, we created two groups: those who cook frozen vegetables (“I cook them by following the instructions on the package”, or “I microwave them”, or “ I cook them on the stove”) and those who consume them raw (“I eat them frozen and raw”, or “I thaw them and eat them without cooking”, or “I add them directly to a blender to make smoothies or juices”). Respondents who reported at least one cooking method, but did not report consuming them raw, were classified into an “only cook” group. Respondents who reported consuming frozen vegetables raw, irrespective of also selecting a cooking method, were classified into an “ever consume raw” group. For analyses examining specific cooking methods that respondents used to prepare frozen vegetables (microwave vs. stove), we excluded respondents who reported only cooking by following instructions without further specifying a method.

To understand and assess risk perceptions of contamination of frozen vegetables with foodborne pathogens, respondents were asked to provide their level of agreement with two statements using a 5-point Likert scale: strongly disagree, disagree, neutral, agree, and strongly agree. The two statements were, “prepackaged frozen vegetables can be contaminated with germs (like *Salmonella, E. coli*, and *Listeria*) that can make people sick” and “prepackaged frozen vegetables need to be fully cooked before consuming them”. For analyses, strongly agree and agree were combined, and strongly disagree and disagree and neutral were combined.

We examined survey responses by demographic characteristics including race/ethnicity, education level, and household income level. We examined responses from respondents who reported having select underlying medical conditions. These underlying medical conditions were chosen for this analysis based upon their availability within the survey dataset and their association with increased risk for severe listeriosis, salmonellosis, or *E. coli* infection, and included cancer, diabetes, and HIV status (U.S. Centers for Disease Control and Prevention, 2019, 2023a). People with cancer were those who self-reported having cancer during the past year. People with diabetes were those who self-reported having diabetes during the past year. Respondents were considered HIV+ if they were ever tested for HIV and they self-reported the result of their most recent HIV test as positive. Respondents with a select underlying medical condition were any respondents with cancer, diabetes, or were HIV positive based on previously defined definitions. We also examined pregnancy status due to the association with severe outcomes, including pregnancy loss associated with listeriosis. Pregnancy included respondents who self-reported their partner and themselves as currently pregnant during completion of the survey.

### Analysis.

Data were weighted by gender, age, household income, race/ethnicity, household size, education, census region, metro status, and parental status of children 11–17 years old to match the 2021 U.S. Census Bureau’s and the U.S. Bureau of Labor Statistics’ Current Population Survey proportions and be nationally representative. We analyzed responses to survey questions by respondent demographic characteristics as well as whether respondents have children, their pregnancy status, and self-reported select underlying medical condition (individually and any select underlying medical condition). Weighted percentages and 95% CIs were calculated and compared using Wald chi-square tests. *P* values <0.05 were considered statistically significant. Estimates with a relative standard error >30% were considered unstable. All weighted analyses were conducted using SAS (version 9.4 SAS Institute), and SAS survey procedures were used to account for the weighted sample.

## Results

### Preparation and consumption of frozen vegetables.

Overall, 3,526 respondents (78.8% response rate) completed the FallStyles survey. Among respondents, 3,521 (99.9%) provided a response when asked how they prepare and/or consume prepackaged frozen vegetables. Of these 3,521 respondents, 3,008 (85.4%) reported at least one method, 62 (1.8%) respondents selected “none of these” when asked how they prepare and/or consume prepackaged frozen vegetables, and 451 (12.8%) reported they do not eat prepackaged frozen vegetables. Among the 3,008 respondents who ate frozen vegetables and reported a specific frozen vegetable preparation method, 2,747 (91.3%) reported only cooking methods while 262 (8.7%) reported ever preparing and/or consuming them raw.

Among the 3,070 respondents who reported eating frozen, prepackaged vegetables, 5.6% reported adding them directly to a blender to make smoothies or juices, 2.7% reported eating them frozen and raw, and 1.9% reported thawing them and then eating them without cooking (Table 1). Respondents who were more likely to report ever preparing and/or consuming frozen vegetables raw were those in the highest household income bracket (≥$75,000 vs. <$75,000) (11.0% vs. 6.0%, *p* < 0.01), the highest education level (bachelor’s degree or higher vs. less than bachelor’s degree) (11.6% vs. 7.1%, *p* < 0.01), and respondents who reported currently having any children under the age of 18 compared with respondents who did not (12.5% vs. 7.4%, *p* < 0.01). Respondents 18–64 years old were more likely than those aged 65 or older to ever consume frozen vegetables raw (10.1% vs. 3.8%, *p* < 0.01). Additionally, respondents without diabetes were more likely to report ever consuming the product raw compared with those with diabetes (9.0% vs. 5.2%, *p* = 0.01). Respondents without a select health condition were also more likely to report ever consuming frozen vegetables raw compared with those reporting a select health condition (11.4% vs 5.6%, *p* < 0.01).

About 41% (40.6%) of respondents who reported eating frozen, prepackaged vegetables, did not report following instructions on the package. Respondents less likely to cook frozen vegetables by following instructions on the package included respondents without cancer compared with respondents with cancer (41.0% vs. 25.4%, *p* < 0.01), and respondents without a select underlying health condition compared with those with a select underlying condition (42.7% vs. 34.1%, *p* ≤ 0.01).

Among 2,360 respondents reporting a specific cooking method, 62.2% reported cooking in the microwave and 69.9% reported cooking them on the stove (Table 1). Respondents more likely to report cooking frozen vegetables on the stove included respondents 18–64 years compared with respondents aged 65 or older (71.8% vs. 62.9%, *p* < 0.01), and respondents not reporting cancer compared with respondents reporting cancer (70.3% vs. 58.1%, *p* = 0.02). Respondents aged 65 or older were more likely than those aged 18–64 to cook frozen vegetables in the microwave (69.9% vs. 60.2%, p < 0.01).

### Risk perceptions.

A total of 3,519 (99.8%) respondents reported their level of agreement with the statement “prepackaged frozen vegetables can be contaminated with germs (like *Salmonella*, *E. coli*, and *Listeria*) that can make people sick”. Among these respondents, 1,199 (34.1%) agreed and 2,320 (65.9%) reported neutral or disagreed (Table 2). Respondents more likely to agree that “frozen vegetables can be contaminated with germs” included males compared with females (39.0% vs. 29.3%, *p* < 0.01), those who completed a bachelor’s degree or higher compared with respondents reporting some college or less (37.9% vs. 32.0%, *p* < 0.01) and respondents reporting currently having a child under the age of 18 compared with those who did not (41.0% vs. 31.7%, *p* < 0.01). Among respondents at higher risk for severe illness, agreement with the statement varied. Respondents reporting diabetes, cancer, and their partner or themselves as currently pregnant did not differ significantly in agreement compared with those not reporting diabetes, not reporting cancer, and not reporting pregnancy themselves or for their partner, respectively. Respondents aged 65 or older were less likely to agree that “frozen vegetables can be contaminated with germs” compared with those 18–64 years (27.4% vs. 35.9%, *p* < 0.01). Similarly, respondents reporting at least one of the select health conditions were less likely to agree that “frozen vegetables can be contaminated with germs” compared with respondents not reporting one of the select health conditions (34.2% vs. 42.2, *p* < 0.01).

A total of 3,523 (99.9%) respondents reported their level of agreement with the statement “prepackaged frozen vegetables need to be fully cooked before consuming them”. Overall, 2,237 (63.5%) agreed, and 1,286 (36.5%) reported neutral or disagreed (Table 3). There were no significant differences in agreement that frozen vegetables need to be cooked between respondents reporting cancer and those who did not, or those reporting any of the three select health conditions and those who did not. Respondents aged 65 or older were more likely to agree that frozen vegetables need to be cooked compared with those aged 18–64 (68.1% vs. 62.2%, *p* < 0.01). Similarly, respondents reporting diabetes were more likely to agree compared with those not reporting diabetes (70.1% vs. 62.4%, *p* < 0.01).

Whether or not respondents agreed that frozen vegetables could be contaminated with “germs (like *Salmonella, E. coli*, and *Listeria*)” was not associated with whether they only cook or ever consume them raw (*p* = 0.25) (Table 4). However, agreement that prepackaged frozen vegetables need to be fully cooked before consuming them was associated with whether they only cook or ever consume them raw (*p* < 0.01). The lack of association between cooking practices and the perception that frozen vegetables can “contain germs”, and the significant association between cooking practices and the perception they need to be cooked, remained among respondents reporting select underlying medical conditions (*p* = 0.98 and *p* < 0.01, respectively) (Table 4).

## Discussion

These findings highlight the importance of reducing contamination of frozen vegetables before they are sold to consumers. Approximately 9% of respondents reported preparing and/or consuming prepackaged frozen vegetables raw. About 40% of respondents did not report following the instructions on the package when preparing frozen vegetables. Only a third of respondents agreed that “prepackaged frozen vegetables can be contaminated with germs”, but 64% agreed prepackaged frozen vegetables need to be fully cooked before consuming them. Perceived risk of contamination was not associated with whether respondents ever consume frozen vegetables raw.

Observations from studies and outbreak investigations demonstrate that raw vegetables can be contaminated with foodborne pathogens (Carstens et al., 2019). Further, a study found that 5.9% of raw vegetables that arrive at frozen food facilities were found to be contaminated with Listeria monocytogenes (Magdovitz et al., 2021). Another study in the United Kingdom sampled frozen vegetables at supermarkets and found *Listeria monocytogenes* in 9.7% of samples, and *E. coli* ≥ 100 cfu/g in 0.5% of samples (Willis et al., 2020). *Listeria monocytogenes, Salmonella*, and STEC bacteria can survive freezing temperatures (Fay et al., 2024). Before the freezing process, vegetables may be blanched, which might be performed as a quality measure, but also frequently reduces the presence of pathogens on the product; however, this depends on the time and temperature of blanching, and may vary by vegetable type (Mazzotta, 2001; Ceylan et al., 2017). Data on the percentage of frozen vegetables sold in the United States that are produced with a blanching step are not available. During the *Listeria* outbreak linked to frozen vegetables in the United States, it is unknown whether the frozen vegetables were blanched (Madad et al., 2023). However, for any *Listeria* or other pathogens remaining on frozen vegetables, growth could have occurred once stored at warmer temperatures, including possibly at retail stores, during transit from a retail store to a consumer’s home, or at a consumer’s home, including thawing to eat them raw. This highlights the importance of controlling contamination of *Listeria* and other foodborne pathogens in frozen vegetables before they reach consumers.

Frozen vegetables are often sold to consumers with the intention that they be cooked, which places responsibility on the consumer for a key component of reducing the risk of illness from consuming a potentially contaminated product. While consumer preparation data for frozen vegetables collected before this survey were limited, there is evidence that some consumers eat them without cooking them. In a 2016 *Listeria* outbreak linked to frozen sweet corn in the United Kingdom, consumption information was available for one patient who was fed not-ready-to-eat, frozen sweet corn pureed with other vegetables (McLauchlin et al., 2021). In FDA’s 2019 Food Safety and Nutrition Survey (FSANS), 10% of respondents reported making dishes using frozen packaged corn without first cooking or heating it (U.S. Food & Drug Administration, 2021). In this survey, nearly 9% of therespondents reporting consuming prepackaged frozen vegetables raw and 40% did not report that they followed the cooking instructions. These data, along with prior research suggesting that decreasing the bacterial load in not ready‐to‐eat, prepackaged frozen vegetables could prevent illnesses (Zoellner et al., 2019), indicate the importance of preventive controls at processing facilities prior to reaching consumers.

Consumers might eat frozen vegetables raw for several reasons. Some frozen vegetables are intended to be consumed frozen, such as frozen vegetables included in a prepackaged smoothie mix, with instructions to add directly to a blender. Understanding whether frozen vegetables are intended to be cooked or can be consumed raw requires reading the package instructions. In our survey, approximately 40% of respondents did not report following the instructions on the package. In the test kitchen meal preparation experiment, only 66% of participants reported reading the label instructions to know how to cook frozen vegetables, while 26% reported prior experience preparing frozen vegetables (Cates et al., 2020). Cooking following prior experience may lead consumers to undercook or not cook frozen vegetables if the prior experience included produce which was intended to be consumed without cooking (i.e., frozen vegetables included in a prepackaged smoothie mix). Further, several blogs, social media platforms and recipes recommend frozen vegetables for teething infants (McCord, 2009; My Connected Motherhood, 2023). Consuming prepackaged frozen vegetables without cooking them may also be out of convenience. For instance, adding frozen vegetables to a dish such as a salad without cooking may save the preparer time. Respondents may not be aware the product is intended to be fully cooked prior to being eaten. In this survey, 37% of respondents either disagreed that prepackaged frozen vegetables need to be fully cooked before consumption or were neutral. In the test kitchen meal preparation experiment, 3% of participants reported they did not need to cook the frozen corn when asked how they knew the corn was done (Cates et al., 2020).

Only one-third of consumers thought frozen vegetables could contain *Salmonella, E. coli*, and *Listeria*. In FDA’s 2019 FSANS survey, 9% of respondents reported that uncooked frozen packaged vegetables, such as packaged frozen spinach or peas, are likely or very likely to “contain bacteria or other germs that could make people sick” (U.S. Food & Drug Administration, 2021). One potential reason respondents may not think prepackaged frozen vegetables can contain pathogens is the limited number of reported outbreaks that have been linked to these products; we queried FDOSS for outbreaks caused by Salmonella, Escherichia, or Listeria linked to frozen foods during 1998–2021, and did not identify any other outbreaks linked to frozen vegetables. In contrast, CDC reported a total of 78 foodborne disease outbreaks linked to leafy greens from 2014 to 2021 (U.S. Centers for Disease Control and Prevention, 2023b). Several of these 78 outbreaks were covered extensively by media, which can impact consumers’ awareness of risk and ultimately their purchasing behaviors (Bitsch & Rombach, 2014). In 2019, FSANS survey data indicated that 44% of respondents had perception that “whole lettuce is likely to contain bacteria or other germs that could make people sick” (U.S. Food & Drug Administration, 2021). Another explanation for the low agreement that “prepackaged frozen vegetables can be contaminated with germs” that we observed could be a perception that freezing kills bacteria. In a survey conducted with participants from the United Kingdom, Norway, and Germany, the mean percentage of respondents agreeing that freezing kills all bacteria was 34% (Veflen & Teixeira, 2022). A survey of U.S. consumers found that only 35% of respondents knew freezing does not kill harmful bacteria which is present on tree nuts (Swinehart, 2023). While freezing inhibits the growth of *Listeria* (U.S. Food & Drug Administration, 2017), a research study conducted in the United Kingdom found *Listeria* was present in 10% of frozen vegetables (Willis et al., 2020).

Respondents in this analysis were more likely to agree that prepackaged frozen vegetables need to be fully cooked before consumption (63.5%) than agree that they can be “contaminated with germs” (34.1%). Further, whether a respondent only cooks prepackaged frozen vegetables or ever consumes them raw was associated with the respondent’s belief that they required cooking, but not their perception of whether they could be “contaminated with germs”. These findings suggest that the perception of contamination and subsequently, risk of illness, may not be a driver in whether respondents only cook or ever consume prepackaged frozen vegetables raw. Understanding why respondents thought frozen vegetables need to be cooked can help food safety professionals frame future prevention strategies and target current knowledge gaps. There are also reasons beyond needing to cook frozen vegetables that could drive consumer preparation practices. One reason consumers might cook their frozen vegetables is taste preference (Hoppu et al., 2021), but further research to characterize and understand the main drivers of cooking practices is warranted. Knowledge of consumers’ beliefs regarding whether a product is expected to be consumed raw vs. requiring cooking, and how this translates to safe handling, is not well researched.

This study is subject to several limitations. These survey questions did not distinguish between types of frozen vegetables, which may be prepared differently. Further research into the types of prepackaged frozen vegetables being consumed raw and how these are being prepared can help food safety professionals prevent future outbreaks. Due to a lack of granularity in the response options, there may be variability within the methods considered “cooking” at the consumer level. For example, respondents reporting cooking on the stove may not be cooking their frozen vegetables thoroughly. Respondents were asked how they prepare their frozen vegetables, but there was no time-frame provided. Therefore, it cannot be assumed people with any of the select underlying health conditions prepared their vegetables as reported during the same period for which they reported having a select, underlying condition. It is unclear whether the prepackaged frozen vegetables that respondents reported consuming raw were considered ready-to-eat by the manufacturer before they were sold to consumers. Cooking instructions may vary across products; therefore, we were unable to examine whether the cooking methods that respondents reported corresponded with the cooking instructions on package labels. Further, comprehensive data on cooking instructions are not readily accessible so we could not determine whether frozen vegetable producers primarily recommend one cooking method over another. We did not assess the potential for cross-contamination and cannot make any assumptions regarding how respondents handle frozen vegetables. For the analyses related to pregnancy, it is unknown whether the person responding to the survey was pregnant or their partner was pregnant, which could influence their responses. Finally, as this is self-reported survey data, it is subject to self-reporting bias.

Frozen vegetables can contribute to a healthy diet, and thus, the safety of this product is particularly essential (Storey & Anderson, 2018). Along with understanding how consumers are preparing frozen vegetables, understanding their perceived risk of foodborne illness from frozen vegetables can inform the development of prevention strategies. We found that although a relatively low proportion of respondents consumed frozen vegetables raw, many did not report cooking them according to package instructions, nor were they aware that frozen vegetables could “contain germs” that could make them sick. Strategies that rely primarily on consumers to cook frozen vegetables to reduce or eliminate pathogens would require educating consumers about the possible risk associated with frozen vegetables, assessing whether consumers understand the difference between ready-to-eat and not-ready-to-eat products, determining whether consumers read and are able to follow label instructions, and addressing barriers to reading and following instructions. These findings show that because some consumers may not be cooking frozen vegetables before eating them, or reading instructions on packaging, preventive controls in the production of frozen vegetables are critically important to reduce or eliminate pathogens prior to reaching the consumer.

## Figures and Tables

**Table 1:** Weighted prevalence estimates of survey respondents’ preparation and consumption of prepackaged, frozen vegetables by selected characteristics – Porter Novelli ConsumerStyles survey, United States, September 2022

Characteristic	I cook them by following the instructions on the package	I eat them frozen and raw	I thaw them and then eat them without cooking	I add them directly to a blender to make smoothies or juices	None of these	I microwave them *N* = 2,360^H^	I cook them on the stove *N* = 2,360^H^
	*Weighted % (95% CI)*	*Weighted % (95% CI)*	*Weighted % (95% CI)*	*Weighted % (95% CI)*	*Weighted % (95% CI)*	*Weighted % (95% CI)*	*Weighted % (95% CI)*
**Overall**	**1,830**	**83**	**57**	**172**	**62**	**1,469**	**1,649**
***N* = 3,070**	**59.6 (57.6–61.6)**	**2.7 (2.0–3.4)**	**1.9 (1.3–2.5)**	**5.6 (4.6–6.7)**	**2.0 (1.4–2.6)**	**62.2 (59.9–64.6)**	**69.9 (67.7–72.0)**
**Age**							
18–29	62.8 (56.7–68.8)	3.3 (0.9–5.7)^±^	2.4 (0.4–4.4)^±^	9.7 (6.0–13.5)	3.4 (1.2–5.7)^±^	61.0 (54.1–67.9)	71.0 (64.7–77.4)
30–44	57.3 (53.0–61.6)	4.1 (2.4–5.9)	2.3 (1.0–3.6)	6.8 (4.6–9.0)	1.4 (0.4–2.3)^±^	58.7 (53.9–63.5)	77.0 (72.9–81.0)
45–64	57.7 (54.4–60.9)	2.2 (1.3–3.2)	1.6 (0.8–2.3)	4.6 (3.2–6.0)	2.4 (1.4–3.5)	60.9 (57.2–64.7)	67.6 (64.1–71.1)
65+	62.3 (59.3–65.3)	1.1 (0.5–1.7)	1.2 (0.6–1.9)	2.0 (1.1–2.9)	1.0 (0.4–1.5)	69.9 (66.5–73.4)	62.9 (59.5–66.3)
**Sex**							
Female	58.7 (55.8–61.6)	3.0 (1.9–4.1)	1.8 (1.0–2.6)	5.9 (4.4–7.4)	1.5 (0.8–2.2)	62.2 (58.9–65.5)	70.4 (67.5–73.4)
Male	60.6 (57.7–63.5)	2.3 (1.4–3.3)	1.9 (1.0–2.8)	5.4 (3.9–6.8)	2.6 (1.5–3.6)	62.3 (58.9–65.6)	69.2 (66.0–72.3)
Prefer to self-describe	67.2 (42.0–92.4)	–	–	–	5.1 (0.0–15.1)^±^	65.6 (36.6–94.6)	80.7 (55.3–100.0)
**Race/Ethnicity**							
American Indian/Alaska Native, non-Hispanic	28.4 (4.2–52.6)^±^	–	–	–	–	75.2 (51.4–99.1)	56.9 (30.4–83.5)
Asian, Hawaiian/Pacific Islander, non-Hispanic	52.4 (42.4–62.4)	1.7 (0.0–4.0)^±^	1.7 (0.0–4.2)^±^	6.1 (1.3–10.9)^±^	2.4 (0.0–5.3)^±^	51.8 (40.5–63.2)	77.7 (67.8–87.5)
Black, non-Hispanic	57.5 (51.0–64.0)	1.0 (0.0–2.3)^±^	0.2 (0.0–0.6)^±^	4.2 (1.9–6.5)	2.6 (0.3–4.8)^±^	43.2 (36.0–50.3)	82.2 (76.6–87.7)
Hispanic	58.9 (52.7–65.1)	3.7 (1.1–6.2)^±^	1.4 (0.0–2.7)^±^	8.4 (4.7–12.1)	0.4 (0.0–0.9)^±^	50.6 (43.2–57.9)	77.4 (71.3–83.4)
White, non-Hispanic	61.1 (58.8–63.4)	3.0 (2.1–3.9)	2.4 (1.5–3.2)	5.1 (3.9–6.3)	2.3 (1.5–3.1)	69.9 (67.4–72.4)	64.5 (61.9–67.0)
Multiple, non-Hispanic	71.8 (61.4–82.1)	0.7 (0.0–2.1)^±^	0.6 (0.0–1.6)^±^	9.1 (3.1–15.0)^±^	0.8 (0.0–2.3)^±^	65.3 (53.1–77.6)	86.8 (79.3–94.3)
**Household Income**							
<$25,000	54.1 (47.8–60.5)	2.4 (0.5–4.3)^±^	0.7 (0.0–1.8)^±^	4.3 (1.7–6.9)^±^	2.4 (0.6–4.2)^±^	56.7 (49.5–63.9)	72.1 (65.8–78.3)
$25,000–49,999	61.7 (56.6–66.7)	3.4 (1.2–5.7)^±^	2.3 (0.5–4.1)^±^	4.1 (1.8–6.3)	2.0 (0.5–3.6)^±^	56.8 (50.9–62.8)	74.2 (69.3–79.2)
$50,000–74,999	59.6 (54.4–64.7)	1.1 (0.1–2.1)^±^	0.9 (0.1–1.7)^±^	2.3 (0.8–3.8)^±^	2.1 (0.2–3.9)^±^	61.2 (55.1–67.3)	69.4 (63.9–74.9)
$75,000+	60.2 (57.6–62.9)	3.0 (2.0–4.0)	2.3 (1.4–3.2)	7.4 (5.8–9.0)	1.9 (1.1–2.6)	65.7 (62.7–68.7)	68.1 (65.2–70.9)
**Education**							
High school grad or less	57.9 (54.2–61.7)	2.3 (1.0–3.7)	2.0 (0.8–3.2)	4.8 (3.0–6.7)	2.5 (1.3–3.7)	56.4 (52.0–60.9)	72.2 (68.3–76.1)
Some college	58.5 (54.7–62.3)	2.3 (1.2–3.5)	1.1 (0.4–1.7)^±^	4.1 (2.5–5.7)	2.0 (0.9–3.1)	63.8 (59.5–68.0)	68.7 (64.6–72.7)
College+	62.2 (59.2–65.2)	3.3 (2.1–4.5)	2.3 (1.3–3.3)	7.6 (5.7–9.4)	1.5 (0.7–2.3)	67.0 (63.6–70.5)	68.4 (65.2–71.6)
**Geographic Region**							
Northeast	60.0 (55.5–64.6)	1.2 (0.2–2.2)^±^	2.7 (1.2–4.3)	6.7 (4.1–9.3)	2.3 (0.8–3.8)^±^	62.2 (57.0–67.4)	70.6 (65.9–75.2)
South	62.6 (59.2–65.9)	3.6 (2.1–5.1)	1.3 (0.3–2.2)^±^	5.1 (3.5–6.8)	1.4 (0.7–2.2)	61.7 (57.7–65.7)	72.3 (68.8–75.8)
Midwest	57.0 (52.6–61.3)	2.5 (1.4–3.7)	1.5 (0.5–2.5)^±^	3.5 (1.8–5.3)	3.0 (1.2–4.9)^±^	66.5 (61.6–71.4)	64.5 (59.8–69.3)
West	56.8 (52.4–61.2)	2.5 (1.1–3.9)	2.4 (1.1–3.8)	7.5 (4.9–10.1)	1.8 (0.8–2.8)	59.3 (54.3–64.3)	70.4 (66.0–74.9)
**Urban/city**							
Urban	57.6 (54.0–61.2)	2.9 (1.6–4.2)	2.2 (1.0–3.3)	6.9 (5.0–8.8)	2.2 (1.1–3.3)	57.7 (53.6–61.7)	74.1 (70.5–77.6)
Suburban	60.8 (57.9–63.7)	2.4 (1.4–3.4)	1.6 (0.9–2.3)	5.5 (3.9–7.0)	2.3 (1.3–3.2)	66.3 (62.9–69.6)	65.7 (62.5–68.9)
Rural	60.3 (55.6–65.0)	3.0 (1.2–4.8)^±^	1.9 (0.3–3.5)^±^	3.5 (1.3–5.6)^±^	0.9 (0.0–1.9)^±^	61.2 (55.5–66.8)	72.3 (67.6–77.0)
**Housing ownership**							
Own	60.4 (58.1–62.7)	2.7 (1.9–3.5)	1.8 (1.1–2.5)	4.6 (3.5–5.7)	1.5 (0.9–2.1)	65.6 (63.0–68.3)	69.0 (66.5–71.5)
Rent	57.0 (52.5–61.4)	2.7 (1.1–4.2)^±^	1.8 (0.6–3.0)^±^	8.4 (5.8–11.0)	2.9 (1.4–4.4)	54.2 (49.2–59.2)	73.1 (68.8–77.4)
Occupied without payment of rent	65.0 (49.0–81.0)	3.9 (0.0–11.3)^±^	3.9 (0.0–11.3)^±^	5.3 (0.0–11.6)^±^	9.8 (0.0–19.9)^±^	52.7 (34.0–71.5)	55.2 (36.7–73.7)
**Currently have any children under the age of 18** ^A^							
Yes	57.3 (52.9–61.7)	5.3 (3.2–7.4)	2.7 (1.1–4.2)	7.4 (5.0–9.7)	1.3 (0.2–2.4)^±^	60.2 (55.2–65.2)	75.5 (71.3–79.8)
No	60.5 (58.2–62.8)	1.8 (1.1–2.4)	1.6 (1.0–2.2)	5.0 (3.9–6.1)	2.2 (1.5–3.0)	62.9 (60.3–65.6)	68.0 (65.5–70.4)
**Youngest Child Age** ^B^							
<1 year	64.6 (40.1–89.2)	2.8 (0.0–8.3)^±^	–	–	–	49.6 (19.1–80.1)^±^	80.1 (58.4–100.0)
1–17 years	57.0 (52.5–61.5)	5.4 (3.2–7.5)	2.8 (1.2–4.4)	7.6 (5.2–10.1)	1.4 (0.2–2.5)^±^	60.5 (55.5–65.6)	75.4 (71.0–79.7)
**My partner and I are currently pregnant** ^C^							
Yes	66.9 (44.2–89.5)	–	–	6.9 (0.0–16.5)^±^	10.8 (0.0–30.1)^±^	64.6 (34.7–94.6)	63.3 (32.2–94.4)
No	59.7 (57.6–61.7)	2.7 (2.0–3.5)	1.9 (1.3–2.5)	5.6 (4.6–6.7)	1.8 (1.2–2.3)	62.6 (60.2–64.9)	70.0 (67.9–72.2)
**Select**^D^ **underlying conditions**							
**Cancer**^E^							
Yes	74.6 (67.6–81.6)	0.8 (0.0–2.3)^±^	2.4 (0.0–5.2)^±^	1.7 (0.0–3.7)^±^	1.4 (0.0–3.5)^±^	72.9 (64.2–81.6)	58.1 (48.4–67.8)
No	59.0 (56.8–61.1)	2.6 (1.9–3.3)	1.7 (1.1–2.3)	5.8 (4.7–6.8)	2.1 (1.4–2.7)	62.1 (59.7–64.5)	70.3 (68.1–72.4)
**Diabetes**^F^							
Yes	64.2 (58.6–69.8)	1.8 (0.2–3.4)^±^	1.0 (0.1–1.9)^±^	3.5 (1.3–5.7)^±^	2.8 (0.8–4.8)^±^	61.1 (54.5–67.7)	68.8 (62.9–74.7)
No	58.9 (56.7–61.1)	2.7 (1.9–3.4)	1.8 (1.2–2.5)	5.9 (4.7–7.1)	1.9 (1.3–2.6)	62.6 (60.1–65.1)	70.0 (67.7–72.3)
**HIV Test**^G^							
Positive	72.2 (30.0–100.0)	27.8 (0.0–70.0)^±^	–	–	–	50.6 (3.5–97.8)^±^	51.8 (4.3–99.3)^±^
Negative	58.5 (54.7–62.3)	3.0 (1.7–4.3)	1.6 (0.6–2.6)^±^	6.9 (5.0–8.7)	1.8 (0.7–3.0)^±^	60.2 (55.9–64.4)	75.1 (71.4–78.7)
**Select condition (Cancer/Diabetes/HIV+)**							
Yes	65.9 (61.1–70.7)	2.2 (0.4–3.9)^±^	1.3 (0.3–2.2)^±^	3.2 (1.4–5.0)	2.6 (0.9–4.3)^±^	62.4 (56.6–68.2)	67.2 (62.0–72.5)
No	57.3 (53.2–61.5)	3.2 (1.8–4.7)	1.7 (0.6–2.8)^±^	7.6 (5.5–9.8)	1.6 (0.4–2.8)^±^	60.2 (55.6–64.8)	75.4 (71.5–79.3)

± Estimate is unstable; relative standard error is >30%.

A 8 respondents refused to answer whether or not they currently had a child under the age of 18.

B Only asked among people who selected “yes” for currently having any children under the age of 18.

C 28 respondents refused to answer.

D Selected conditions based on the availability of data and relevance to severity of *Listeria*.

E 47 respondents refused to answer.

F 47 respondents refused to answer.

G Only asked among those who replied “yes” to ever having been tested for HIV. Options included: 1. Negative 2. Positive 3. Indeterminate 4. Never obtained results 5. I don’t know 6. Prefer not to answer.

H Removed respondents who selected “I cook them by following instructions on the package” but did not select any other preparation method, from the calculation.

**Table 2:** Demographic characteristics of respondents who agree and disagree prepackaged frozen vegetables can contain germs that can make people sick–Porter Novelli ConsumerStyles survey, United States, September 2022.

Characteristic	Total *N* = 3,519	Agree *N* = 1,199 34.1 (32.2–35.9)	Neutral/Disagree *N* = 2,320 65.9 (64.1–67.8)	*P* value
	*Weighted N*	*Weighted % (95% CI)*	*Weighted % (95% CI)*	
**Age**				
18–29	684	32.6 (27.2–38.0)	67.4 (62.0–72.8)	0.09
30–44	925	41.5 (37.5–45.5)	58.5 (54.5–62.5)	<0.01
45–64	1,135	33.4 (30.5–36.3)	66.6 (63.7–69.5)	<0.01
65+	774	27.4 (24.8–30.0)	72.6 (70.0–75.2)	Ref.
**Sex**				
Female	1,806	29.3 (26.8–31.8)	70.7 (68.2–73.2)	<0.01
Male	1,700	39.0 (36.3–41.7)	61.0 (58.3–63.7)	Ref.
**Race/Ethnicity**				
American Indian/Alaska Native, non-Hispanic	34	21.8 (2.3–41.3)^±^	78.2 (58.7–97.7)	0.30
Asian, Hawaiian/Pacific Islander, non-Hispanic	218	41.8 (32.7–50.9)	58.2 (49.1–67.3)	0.06
Black, non-Hispanic	421	41.3 (35.2–47.4)	58.7 (52.6–64.8)	<0.01
Hispanic	588	31.6 (26.3–36.9)	68.4 (63.1–73.7)	0.72
Multiple races, non-Hispanic	51	40.2 (29.0–51.4)	59.8 (48.6–71.0)	0.20
White, non-Hispanic	2,206	32.6 (30.5–34.7)	67.4 (65.3–69.5)	Ref.
**Household Income**				
<$25,000	449	34.9 (29.2–40.7)	65.1 (59.3–70.8)	0.99
$25,000–49,999	595	35.1 (30.4–39.8)	64.9 (60.2–69.6)	0.95
$50,000–74,999	577	29.3 (24.9–33.8)	70.7 (66.2–75.1)	0.03
$75,000+	1,898	35.0 (32.5–37.4)	65.0 (62.6–67.5)	Ref.
**Education**				
High school grad or less	1,325	31.4 (28.1–34.7)	68.6 (65.3–71.9)	<0.01
Some college	954	32.8 (29.4–36.1)	67.2 (63.9–70.6)	0.02
College+	1,239	37.9 (35.1–40.8)	62.1 (59.2–64.9)	Ref.
**Currently have any children under the age of 18** ^A^				
Yes	881	41.0 (36.8–45.1)	59.0 (54.9–63.2)	<0.01
No	2,630	31.7 (29.6–33.7)	68.3 (66.3–70.4)	Ref.
**My partner and I are currently pregnant**				
Yes	34	42.8 (21.0–64.5)	57.2 (35.5–79.0)	0.44
No	3,456	34.1 (32.3–36.0)	65.9 (64.0–67.7)	Ref.
**Select**^B^ **underlying health conditions**				
**Cancer**				
Yes	123	30.0 (23.0–37.0)	70.0 (63.0–77.0)	0.28
No	3,348	34.0 (32.1–35.9)	66.0 (64.1–67.9)	Ref.
**Diabetes**				
Yes	394	35.3 (30.2–40.4)	64.7 (59.6–69.8)	0.56
No	3,078	33.7 (31.7–35.7)	66.3 (64.3–68.3)	Ref.
**HIV Test**^C^				
Positive	10	44.7 (7.6–81.7)^±^	55.3 (18.3–92.4)^±^	0.92
Negative	1,015	42.8 (39.3–46.4)	57.2 (53.6–60.7)	Ref.
**≥1 Select underlying health condition (Cancer/Diabetes/HIV+)**				
Yes	493	34.2 (29.8–38.6)	65.8 (61.4–70.2)	<0.01
No	863	42.2 (38.3–46.1)	57.8 (53.9–61.7)	Ref.

Reference groups for age and race/ethnicity were selected using the percentage of people that consumed frozen vegetables in the past 7 days from the Foodborne Diseases Active Surveillance Network (FoodNet) Population Survey (FoodNet Fast|CDC). The reference group for age was 65 years or older (63.2%), and for race/ethnicity, was White, Non-Hispanic (59.9%). Additionally, as adults 65 years and older have an increased risk for becoming very sick from food poisoning, this age group was compared with other age groups (U.S. Centers for Disease Control and Prevention, 2022a). The reference group for education level and income were selected based on studies that identify groups more likely to practice risky food safety behaviors (Patil et al., 2005; Cates et al., 2009).

± Estimate is unstable; relative standard error is >30%.

A 7 respondents refused to answer whether they currently have children under the age of 18.

B Selected conditions based on availability of data and relevance to severity of *Listeria*.

C Only asked among those who replied “yes” to ever having been tested for HIV.

**Table 3:** Demographic characteristics of consumers who agree and disagree prepackaged frozen vegetables need to be cooked before consumption – Porter Novelli ConsumerStyles survey, United States, September 2022

Characteristic	Total *N* = 3,523	Agree *N* = 2,237 63.5 (61.6–65.4)	Neutral/Disagree *N* = 1,286 36.5 (34.6–38.4)	*P* value
	*Weighted N*	*Weighted % (95% CI)*	*Weighted % (95% CI)*	
**Age**				
18–29	686	60.7 (55.1–66.4)	39.3 (33.6–44.9)	0.02
30–44	927	63.9 (59.9–67.8)	35.1 (32.2–40.1)	0.08
45–64	1,136	61.7 (58.7–64.6)	38.3 (35.4–41.3)	<0.01
65+	774	68.1 (65.5–70.8)	31.9 (29.2–34.5)	Ref.
**Sex**				
Female	1,807	61.8 (59.1–64.5)	38.2 (35.5–40.9)	0.05
Male	1,704	65.5 (62.8–68.1)	34.5 (31.9–37.2)	Ref.
**Race/Ethnicity**				
American Indian/Alaska Native, non-Hispanic	34	64.0 (38.6–89.4)	36.0 (10.6–61.4)^±^	0.90
Asian, Hawaiian/Pacific Islander, non-Hispanic	218	65.9 (57.1–74.6)	34.1 (25.4–42.9)	0.44
Black, non-Hispanic	421	73.3 (67.7–79.0)	26.7 (21.0–32.3)	<0.01
Hispanic	588	60.2 (54.6–65.8)	39.8 (34.2–45.4)	0.49
Multiple races, non-Hispanic	51	60.8 (50.1–71.5)	39.2 (28.5–49.9)	0.78
White, non-Hispanic	2,211	62.3 (60.2–64.5)	37.7 (35.5–39.8)	Ref.
**Household Income**				
<$25,000	450	64.1 (58.4–69.7)	35.9 (30.3–41.6)	0.56
$25,000–49,999	597	67.8 (63.3–72.3)	32.3 (27.7–36.7)	0.04
$50,000–74,999	577	62.8 (58.0–67.6)	37.2 (32.4–42.0)	0.83
$75,000+	1,900	62.2 (59.7–64.7)	37.8 (35.3–40.3)	Ref.
**Education**				
High school grad or less	1,328	63.8 (60.4–67.3)	36.2 (32.7–39.6)	0.69
Some college	955	63.8 (60.3–67.2)	36.2 (32.8–39.7)	0.72
College+	1,240	62.9 (60.1–65.8)	37.1 (34.2–39.9)	Ref.
**Currently have any children under the age of 18** ^A^				
Yes	883	62.9 (58.8–67.1)	37.1 (32.9–41.2)	0.78
No	2,633	63.6 (61.5–65.7)	36.4 (34.3–38.5)	Ref.
**My partner and I are currently pregnant**				
Yes	34	74.0 (53.1–95.0)	26.0 (5.0–46.9)^±^	0.32
No	3,461	63.5 (61.6–65.4)	36.5 (34.6–38.4)	Ref.
**Select**^B^ **underlying health conditions**				
**Cancer**				
Yes	124	62.1 (55.0–69.3)	37.9 (30.7–45.0)	0.76
No	3,352	63.3 (61.4–65.3)	36.7 (34.7–38.6)	Ref.
**Diabetes**				
Yes	399	70.1 (65.2–75.1)	29.9 (24.9–34.8)	<0.01
No	3,078	62.4 (60.3–64.4)	37.6 (35.6–39.7)	Ref.
**HIV Test**^C^				
Positive	10	64.8 (26.1–100)^±^	35.2 (0.0–73.9)^±^	0.89
Negative	1,015	67.6 (64.3–70.9)	32.4 (29.1–35.7)	Ref.
**≥1 Select underlying health condition (Cancer/Diabetes/HIV+)**				
Yes	498	67.8 (63.5–72.1)	32.3 (27.9–36.5)	0.78
No	863	67.0 (63.4–70.6)	33.0 (29.4–36.6)	Ref.

Reference groups for age and race/ethnicity were selected using the percentage of people that consumed frozen vegetables in the past 7 days from the Foodborne Diseases Active Surveillance Network (FoodNet) Population Survey (FoodNet Fast | CDC). The reference group for age was 65 years or older (63.2%), and for race/ethnicity, was White, Non-Hispanic (59.9%). Additionally, as adults 65 years and older have an increased risk for becoming very sick from food poisoning, this age group was compared with other age groups (U.S. Centers for Disease Control and Prevention, 2022a). The reference group for education level and income were selected based on studies that identify groups more likely to practice risky food safety behaviors (Patil et al., 2005; Cates et al., 2009).

± Estimate is unstable; relative standard error is > 30%.

A 7 respondents refused to answer whether they currently have children under the age of 18.

B Selected conditions based on availability of data and relevance to severity of *Listeria*.

C Only asked among those who replied “yes” to ever having been tested for HIV.

**Table 4:** Relationship between consumer preparation methods and risk perceptions, United States, September 2022

	All	Only Cook	Ever Consume Raw	*P* value
	*Weighted n*	*Weighted % (95% CI)*	*Weighted % (95% CI)*	
**All**	*n* = 3,008	*n* = 2,747 91.3 (90.0–92.6)	*n* = 262 8.7 (7.4–10.0)	
Prepackaged frozen vegetables can be contaminated with germs (like *Salmonella*, *E. coli*, and *Listeria*) that can make people sick.	*n* = 3,002	*n* = 2,740	*n* = 262	
Agree	35.0 (32.9–37.0)	34.6 (32.5–36.6)	39.1 (31.6–46.6)	0.25
Neutral/Disagree	65.0 (63.0–67.1)	65.4 (63.4–67.5)	60.9 (53.4–68.4)	–
Prepackaged frozen vegetables need to be fully cooked before consuming them.	*n* = 3,006	*n* = 2,745	*n* = 262	
Agree	65.4 (63.4–67.4)	67.6 (65.5–69.6)	42.7 (35.2–50.3)	<0.01
Neutral/Disagree	34.6 (32.6–36.6)	32.4 (30.4–34.5)	57.3 (49.7–64.8)	–
**Pregnant**				
Prepackaged frozen vegetables can be contaminated with germs (like *Salmonella*, *E. coli*, and *Listeria*) that can make people sick.	*n* = 28	*n* = 26	*n* = 2	
Agree	42.5 (20.5–64.6)	37.7 (15.0–60.4)^±^	100 (100–100)	–
Neutral/Disagree	57.5 (35.4–79.5)	62.3 (39.6–85.0)	–	–
Prepackaged frozen vegetables need to be fully cooked before consuming them.	*n* = 28	*n* = 26	*n* = 2	
Agree	80.5 (63.0–98.1)	78.9 (60.0–97.8)	100 (100–100)	–
Neutral/Disagree	19.5 (1.9–37.0)	21.1 (2.2–40.0)^±^	–	–
**Cancer/Diabetes/HIV Test +**				
Prepackaged frozen vegetables can be contaminated with germs (like *Salmonella*, *E. coli*, and *Listeria*) that can make people sick.	*n* = 419	*n* = 396	*n* = 24	
Agree	34.4 (29.6–39.2)	33.9 (29.0–38.8)	42.7 (19.7–65.6)	0.48
Neutral/Disagree	65.6 (60.8–70.4)	66.1 (61.2–71.0)	57.3 (34.4–80.3)	–
Prepackaged frozen vegetables need to be fully cooked before consuming them.	*n* = 424	*n* = 401	*n* = 24	
Agree	68.7 (64.0–73.4)	70.8 (66.1–75.5)	33.4 (11.9–54.8)^±^	<0.01
Neutral/Disagree	31.3 (26.6–36.0)	29.2 (24.5–33.9)	66.6 (45.2–88.1)	–
**65 +**				
Prepackaged frozen vegetables can be contaminated with germs (like *Salmonella*, *E. coli*, and *Listeria*) that can make people sick.	*n* = 677	*n* = 652	*n* = 26	
Agree	27.2 (24.5–30.0)	27.2 (24.4–30.1)	27.1 (13.9–40.2)	0.98
Neutral/Disagree	72.8 (70.0–75.5)	72.8 (69.9–75.6)	72.9 (59.8–86.1)	–
Prepackaged frozen vegetables need to be fully cooked before consuming them.	*n* = 678	*n* = 652	*n* = 26	
Agree	69.5 (66.7–72.3)	70.4 (67.5–73.3)	46.8 (31.3–62.3)	<0.01
Neutral/Disagree	30.5 (27.7–33.3)	29.6 (26.7–32.5)	53.2 (37.7–68.7)	–

± Estimate is unstable; relative standard error is >30%.
